# Haploidentical vs matched sibling donor transplant for paroxysmal nocturnal haemoglobinuria: A multicenter study

**DOI:** 10.1038/s41408-022-00682-w

**Published:** 2022-06-24

**Authors:** Limin Liu, Shunqing Wang, Erlie Jiang, Yanming Zhang, Jianyong Li, Yuewen Fu, Meiqing Lei, Kailin Xu, Mingzhen Yang, Yinghao Lu, Miao Miao, Depei Wu

**Affiliations:** 1 National Clinical Research Center for Hematologic Diseases, The First Affiliated Hospital of Soochow University, Jiangsu Institute of Hematology, Key Laboratory of Thrombosis and Hemostasis of Ministry of Health, Collaborative Innovation Center of Hematology, Suzhou, China; 2 Department of Hematology, Guangzhou First People’s Hospital, Guangzhou Medical University, Guangzhou, China; 3 State Key Laboratory of Experimental Hematology, National Clinical Research Center for Blood Diseases, Institute of Hematology & Blood Diseases Hospital, Chinese Academy of Medical Sciences & Peking Union Medical College, Tianjin, China; 4 Department of hematology, The Affiliated Huai’an Hospital of Xuzhou Medical University and The Second People’s Hospital of Huai’an, Huai’an, China; 5 Department of hematology, The First Affiliated Hospital of Nanjing Medical University, Nanjing, China; 6 Department of hematology, Affiliated Cancer Hospital Zhengzhou University, Henan Tumor Hospital, Institute of Hematology, Zhengzhou, China; 7 Department of hematology, Haikou Municipal People’s Hospital, Affiliated Haikou Hospital Xiangya School of Medicine Central South University, Haikou, China; 8 Department of hematology, The Affiliated Hospital of Xuzhou Medical University, Xuzhou, China; 9 Department of hematology, The Fourth Affiliated Hospital of Anhui Medical University, Hefei, China; 10 Department of hematology, The Affiliated Hospital of Guizhou Medical University, Guiyang, China

**Keywords:** Anaemia, Haematopoietic stem cells

Dear Editor,

Paroxysmal nocturnal haemoglobinuria (PNH)) is a rare, progressive, and life-threatening hematopoietic stem cell disorder, with the current worldwide prevalence estimated at 12 to 13 cases per million [1–3]. Allogeneic haematopoietic stem cell transplantation (allo-HSCT) should be recommended in PNH patients with severe aplastic anaemia and the presence of a PNH clone, with evidence of clonal evolution such as the myelodysplastic syndrome (MDS) or leukaemia [4]. Eculizumab is an anti-C5 monoclonal antibody and its use in patients with PNH has significantly changed the management and clinical outcomes of the disease [4–7]. Currently, although eculizumab is used widely to treat patients with PNH, an allo-HSCT is still the only curative therapy for PNH, with a HLA-matched sibling donor HSCT (MSD-HSCT) being the first treatment option. Unfortunately, only a small number of patients have a matched sibling. However, alternative sources of stem cells have been reported and MSD-HSCT has similar outcomes to those of an unrelated donor HSCT (UD-HSCT) in patients with PNH [8]. The immediate availability of a suitable haploidentical donor (HID) for the majority of patients within an appropriate time frame is a clear advantage. However, there are only a small number of reports on the use of HID-HSCT for treating PNH [9]. In previous studies, we reported the outcomes of PNH patients who underwent HID-HSCT at our center and obtained encouraging results [10–13]. The multicenter study described in this paper retrospectively compared 73 cases who had a HID-HSCT with 78 patients who had a MSD-HSCT between December 2002 and May 2021.

All 167 patients with PNH who underwent an allo-HSCT at our centers between December 2002 and May 2021 were enrolled in this study. Of these 167 patients, 78 were treated by MSD-HSCT, with 16 patients having an UD-HSCT and 73 cases undergoing HID-HSCT. This study was approved by the Ethics Committee of every center in the study. All patients provided written, informed consent before the commencement of therapy. The details of the diagnosis of PNH, eligibility for allo-HSCT, graft collection and infusion, graft-versus-host disease (GVHD) prophylaxis and treatment strategy, definitions and post-transplantation evaluations, supportive care, and post-transplantation surveillance were in line with our previous report [14].

The statistical analyses were conducted on data available from the date of treatment to the final date of patient follow-up (i.e., September 30, 2021). The patient characteristics were compared using the chi-square test and the nonparametric test for continuous variables. The cumulative incidence of GVHD was estimated using the competing risk model, with death as the competing event. The probabilities of overall survival (OS) and GVHD-free and failure-free survival (GFFS) were estimated from the time of treatment using the Kaplan–Meier method, with comparisons of the different patient groups carried out using the log-rank test. For multivariate analysis, the Cox proportional hazard regression model was used to analyze OS, GFFS, and GVHD. The statistical analyses were performed using SPSS version 22.0 (SPSS, Chicago, IL, U.S.A). All *P* values were two-sided and the results were considered statistically significant when the *P* value was < 0.05.

The data for all patient and donor characteristics at the time of transplantation are shown in Table 1. There was no difference between the two groups for the median age of patients and donors, gender, median disease duration, donor–recipient sex match, and blood types of the donor to recipient (*P* > 0.05). In the MSD group, the proportion of patients with classical PNH was higher than in the HID group (*P* = 0.019), whereas the proportion of PNH-AA syndrome patients was lower than in the HID group (*P* = 0.009). The patients received various treatments before transplantation including steroids, androgens, cyclosporine, antithymocyte immunoglobulin (ATG), and growth factors.

**Table 1:** Characteristics of PNH patients and donors.

Variable	HID (*n* = 73)	MSD (*n* = 78)	*P*
Clinical characteristics			
Median age, years (range)	23 (6–54)	30 (14–50)	0.100
≤ 20 years, no. (%)	20 (27.40)	11 (14.10)	0.039
21–39 years, no. (%)	34 (46.58)	46 (58.97)	0.127
≥ 40 years, no. (%)	19 (26.03)	21 (26.92)	0.901
Gender (male/female)	42/31	52/26	0.247
Classification of PNH at transplantation, no. (%)			
Classical PNH	13 (17.81)	27 (34.62)	0.019
PNH in the setting of another BM disorder			
PNH-AA syndrome	59 (80.82)	48 (61.54)	0.009
PNH-MDS	0 (0.00)	3 (3.85)	0.267
PNH-AML	1 (1.37)	0 (0.00)	0.483
Median time from diagnosis to transplantation, months (range)	6 (1–120)	8 (1–360)	0.350
Donor median age, years (range)	34 (11–57)	32 (10–57)	0.482
Donor–recipient sex match, no. (%)			
Male–male	28 (38.36)	28 (35.90)	0.755
Male–female	20 (27.40)	16 (20.51)	0.321
Female–male	14 (19.18)	24 (30.77)	0.101
Female–female	11 (15.07)	10 (12.82)	0.690
Donor–recipient relationship, no. (%)			
Mother–child	11 (15.07)	--	--
Father–child	21 (28.77)	--	--
Child–mother	5 (6.85)	--	--
Child–father	9 (12.33)	--	--
Siblings	27 (36.99)	78 (100.00)	<0.0001
Blood types of donor to recipient, no. (%)			
Matched	45 (61.64)	42 (53.85)	0.333
Major mismatched	10 (13.70)	12 (15.38)	0.769
Minor mismatched	15 (20.55)	17 (21.79)	0.851
Major and minor mismatched	3 (4.11)	7 (8.97)	0.382
Conditioning regimen			
FLU+CY+ATG	7 (9.59)	24 (30.77)	0.001
BU+CY(1)+ATG	46 (64.38)	30 (42.31)	0.003
BU+CY(2)	1 (1.37)	3 (3.85)	0.660
BU+CY+FLU+ATG	6 (8.22)	11 (14.10)	0.253
FLU+BU+ATG	2 (2.74)	5 (6.41)	0.494
FLU+CY+TBI+ATG	11 (15.07)	2 (2.56)	0.006
BU+CY+TBI+ATG	0 (0.00)	1 (1.28)	1.000
CY+ATG	0 (0.00)	2 (2.56)	0.497
GVHD prophylaxis			
CsA	7 (9.59)	28 (35.90)	<0.001
CsA+MTX	2 (2.74)	16 (20.51)	0.001
CsA+MMF+MTX	53 (72.60)	34 (43.59)	<0.001
PTCY	11 (15.07)	0 (0.00)	<0.001
Source of graft, no. (%)			
BM+PB cell	52 (72.22)	39 (50.00)	0.008
BM	2 (2.78)	2 (2.56)	1.000
PB cell	18 (25.00)	37 (47.44)	0.004
Median mononuclear cells, ×10^8^/kg (range)	10.76 (3.96–33.40)	10.44 (4.43–22.00)	0.248
Median CD34^+^ cells, ×10^6^/kg (range)	3.85 (0.54–14.40)	3.47 (1.09–34.10)	0.161
Median neutrophil recovery, days (range)	12 (9–37)	12 (6–24)	0.284
Median platelet recovery, days (range)	15 (7–75)	13 (8–150)	0.280
Delayed platelet recovery, no. (%)	5 (7.35)	7 (8.97)	0.722
Failed plated engraftment, no. (%)	5 (7.35)	4 (5.13)	0.832
Primary graft failure, no. (%)	1 (1.47)	0 (0.00)	0.466
Secondary graft failure, no. (%)	1 (1.47)	1 (1.28)	1.000
Relapse, no. (%)	0 (0.00)	1 (1.28)	1.000
Causes of death, no. (%)			
Primary graft failure	1 (1.37)	0 (0.00)	0.483
Secondary graft failure	1 (1.37)	1 (1.28)	1.000
GVHD	2 (2.74)	0 (0.00)	0.232
Infection	3 (4.11)	5 (6.41)	0.789
Cerebral hemorrhage	4 (5.48)	0 (0.00)	0.112
Thrombotic microangiopathy	2 (2.74)	1 (1.28)	0.954
PTLD	1 (1.37)	0 (0.00)	0.483
Renal *failure*	0 (0.00)	1 (1.28)	1.000
Relapse	0 (0.00)	1 (1.28)	1.000
Median follow-up time among living patients, months (range)	31 (4–110)	26 (4–252)	0.734

*PNH* paroxysmal nocturnal hemoglobinuria; *HID* haploidentical donor; MSD, matched sibling donor; *BM* bone marrow; *AA* aplastic anemia; *MDS* myelodysplastic syndrome; *AML* acute myelogenous leukemia; *FLU* Fludarabine; *CY* cyclophosphamide; *ATG* antithymocyte immunoglobulin; *BU* busulfan; *TBI* total body irradiation; *GVHD* graft-versus-host disease; *CsA* cyclosporin A; *MTX* methotrexate; *MMF* mycophenolate mofetil; *PTCY* post-transplant cyclophosphamide; *PB* peripheral blood; *PTLD* post-transplantation lymphoproliferative diseases. The FLU+CY+ATG: FLU, 30 mg/m^2^/day intravenously (i.v.) on day −7 to −2; CY, 50 mg/kg/day i.v. on day −4 to −3; and ATG, (rabbit, Thymoglobuline®, Genzyme, Cambridge, MA, USA), 2.5 mg/kg/day i.v. on day −8 to −4. The FLU+CY+TBI+ATG: FLU at 30 mg/m^2^/day i.v. on day −5 to −2; CY at 40 mg/kg/day i.v. on days −7 to −6 and days +3 to +4; TBI at 3 Gy on day −1; and ATG at 2 mg/kg/day i.v. on days −5 to −3. The BU+CY(1)+ATG: BU at 3.2 mg/kg/day i.v. on days −7 and −6; CY at 50 mg/kg/day i.v. on days −5 to −2; and ATG at 2.5 mg/kg/day i.v. on days −5 to −2. The BU+CY+FLU+ATG: BU at 3.2 mg/kg/day i.v. on days −8 and −6; CY at 40 mg/kg/day i.v. on days −3 to −2; FLU at 30 mg/m^2^/day i.v. on day −5 to −3; and ATG at 2.5 mg/kg/day i.v. on days −5 to −2. The FLU+BU+ATG: FLU at 30 mg/m^2^/day i.v. on day −7 to −2; BU at 3.2 mg/kg/day i.v. on days −3 and −2; and ATG at 2.5 mg/kg/day i.v. on days −5 to −2. The BU+CY+TBI+ATG: BU at 3.2 mg/kg/day i.v. on days −8 and −6; CY at 40 mg/kg/day i.v. on days −3 to −2; TBI at 3 Gy on day −1; and ATG at 2.5 mg/kg/day i.v. on days −5 to −2. The CY+ATG: CY at 50 mg/kg/day i.v. on days −5 to −2; and ATG at 5 mg/kg/day i.v. on days −5 to −1. PNH-MDS and PNH-AML were used in the BU+CY(2): simustine (Me-CCNU) 250 mg/m^2^/day po on day −10; hydroxycarbamide 40 mg/m^2^/12 h po on day −10; cytarabine 2 g/m^2^/12 h i.v. on days −9 to −8; BU at 3.2 mg/kg/day i.v. on days −7 and −5; CY at 1.8 g/m^2^/day i.v. on days −4 to −3; and ATG at 2.5 mg/kg/day i.v. on days −5 to −2 (HID patients).

In the HID group, 68 evaluable patients survived for more than 30 days and all patients achieved myeloid engraftment with complete chimerism (> 95%) at a median of 12 days (range, 9–37). In the MSD group, all 78 cases survived for more than 30 days and all patients achieved myeloid engraftment with complete chimerism at a median of 12 days (range, 6–24) (*P* = 0.284). The cumulative incidence of 30-day engraftment was 97.10 ± 2.02% and 100.00 ± 0.00% in the HID and MSD groups, respectively (*P* = 0.330) (Fig. 1A). The median time to platelet recovery was 15 days (range, 7–75) in the HID group and 13 days (range, 8–150) in the MSD group (*P* = 0.280). Delayed platelet recovery was demonstrated in 5 patients in the HID group and 7 patients in the MSD group (*P* = 0.722), while failed plated engraftment was observed in 5 patients in the HID group and 4 patients in the MSD group (*P* = 0.832). There was a similar cumulative incidence of platelet engraftment in the HID and MSD groups (92.07 ± 3.56% vs. 97.69 ± 2.14%, respectively, *P* = 0.209) (Fig. 1B). One patient experienced primary graft failure in the HID group whereas no patient had this failure in the MSD group (*P* = 0.466). One patient in both the HID and MSD groups experienced secondary graft failure (*P* = 1.000).

**Fig. 1:**
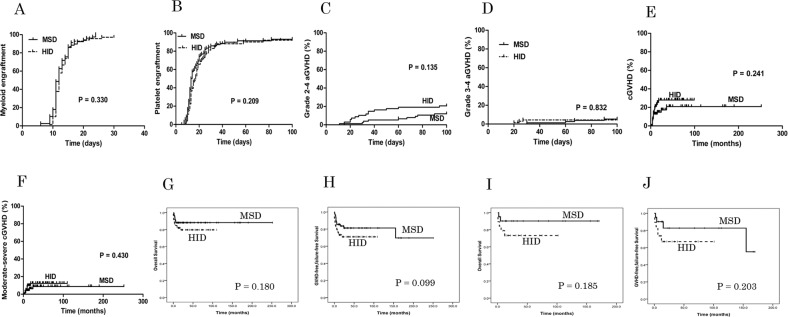
The cumulative incidence of engraftment, graft-versus-host disease (GVHD), overall survival (OS) and GVHD failure-free survival (GFFS). **A** The cumulative incidence of 30-day engraftment was 97.10% ± 2.02% and 100.00 %± 0.00% in the HID and MSD groups, respectively (*P* = 0.330). **B** The cumulative incidence of platelet engraftment was 92.07% ± 3.56% and 97.69% ± 2.14% in the HID and MSD groups (*P* = 0.209). **C** The cumulative incidence of grade 2–4 aGVHD on day +100 was 20.59% ± 4.90% and 11.92% ± 3.73% after the HID and MSD transplants, respectively (*P* = 0.135). **D** The cumulative incidence of grade 3-4 aGVHD on day +100 was 4.39% ± 2.48% and 5.30% ± 2.58% after the HID and MSD transplants, respectively (*P* = 0.832). **E** The cumulative incidence of cGVHD was 27.96 %± 6.11% and 20.86% ± 5.86% in the HID and MSD groups, respectively (*P* = 0.241). **F** The cumulative incidence of moderate-severe cGVHD was 12.31% ± 4.45% and 8.73% ± 3.88% (*P* = 0.430). **G** The probability of three-year OS was 79.7% ± 4.9% and 88.2% ± 3.7% after the HID and MSD transplants, respectively (*P* = 0.180). H) The probability of three-year GFFS was 71.0% ± 5.6% after a HID transplant and 81.2% ± 4.9% after a MSD transplant (*P* = 0.099). **I** In patients ≥ 40 years old, there was no difference in estimated three-year OS between the HID and MSD groups (73.3% ± 10.2% vs. 90.2% ± 6.6%, respectively *P* = 0.185). **J** In patients ≥ 40 years old, there was no difference in estimated three-year GFFS between the HID and MSD groups (67.0% ± 11.2% vs. 82.7% ± 9.4%, *P* = 0.203).

The cumulative incidence of grade 2–4 aGVHD on day +100 was 20.59% ± 4.90% and 11.92% ± 3.73% after the HID and MSD transplants, respectively (*P* = 0.135) (Fig. 1C). The cumulative incidence of grade 3–4 aGVHD on day +100 was 4.39% ± 2.48% and 5.30% ± 2.58% after the HID and MRD transplants, respectively (*P* = 0.832) (Fig. 1D). Multivariate analysis showed that no factors had a significant association with grade 2–4 aGVHD or grade 3–4 aGVHD (*P* > 0.05) (Supplementary Table S1).

Sixty-five patients in the HID group and 74 patients in the MSD group survived for longer than 100 days after transplantation and were used to calculate the incidence of cGVHD. The cumulative incidence of cGVHD was 27.96% ± 6.11% and 20.86% ± 5.86% in the HID and MSD groups, respectively (*P* = 0.241) (Fig. 1E), while the corresponding cumulative incidence of moderate-severe cGVHD was 12.31% ± 4.45% and 8.73% ± 3.88% (*P* = 0.430) (Fig. 1F). Multivariate analysis demonstrated that no factor showed a significant association with either cGVHD or moderate-severe cGVHD (*P* > 0.05) (Table S1).

The median follow-up time in living patients in the HID group was 31 months (range, 4–110) and 26 months (range, 4–252) in the MSD group (*P* = 0.734). During the follow-up period, the TRM rate was 19.19% ± 4.89% in the HID group and 10.50% ± 3.52% in the MSD group (*P* = 0.167). As shown in Table 1, there was no difference in the causes of TRM between the two groups. No patient in the HID group relapsed during the follow-up period, although one PNH-MDS patient in the MSD group relapsed during this period (*P* = 1.000). The probability of three-year OS was 79.7% ± 4.9% and 88.2% ± 3.7% after the HID and MSD transplants, respectively (*P* = 0.180) (Fig. 1G). The probability of three-year GFFS was 71.0% ± 5.6% after a HID transplant and 81.2% ± 4.9% after a MSD transplant (*P* = 0.099) (Fig. 1H). In patients ≥ 40 years old, there was no difference in estimated three-year OS between the HID and MSD groups (73.3% ± 10.2% vs. 90.2% ± 6.6%, respectively *P* = 0.185) (Fig. 1I); there was a similar estimated 3-year GFFS in the two groups (67.0% ± 11.2% vs. 82.7% ± 9.4%, *P* = 0.203) (Fig. 1J). Multivariate analysis identified no factors that showed a significant association with OS and GFFS (*P* > 0.05) (Table S1).

To our knowledge, this study is the first formal comparison of allo-HSCT transplantation using either HID or MSD in PNH patients. Although the study was not prospective or randomized, its strengths include that it was carried out in multiple centers in a relatively large number of patients with this rare disorder. The comparison also provided the opportunity to investigate the currently undefined role of HID-HSCT as the therapy of choice for PNH patients without a suitable MSD who were recommended to receive an allo-HSCT. The study is the first to demonstrate that HID-HSCT has similar TRM, engraftment, OS, GFFS, relapse, and GVHD as that observed in the MSD group.

Although eculizumab has significantly changed the management and clinical outcomes of PNH it is not currently available in China and some other countries. Transplantation is therefore the only curative therapy for PNH, although patients with classical PNH should not be offered a HSCT as initial therapy given the risks of transplant-related morbidity and mortality [8, 15]. The exception to this exclusion are PNH patients living in countries where eculizumab is not available. Patients meeting the criteria for severe aplastic anaemia with PNH clones continue to be suitable candidates for HSCT if they are young and have a suitable donor [14]. In a considerable number of PNH patients, a suitable matched donor is not available or cannot be identified within a reasonable time frame. Although a matched unrelated donor HSCT (MUD-HSCT) has similar outcomes to a MSD-HSCT in patients with PNH [8], the often long period of time taken to identify a matched, unrelated donor may result in disease progression prior to treatment. Therefore, HID-HSCT virtually ensures the opportunity for nearly all patients to benefit from a HSCT and offers the advantage of immediate accessibility to transplantation therapy. Currently, there are only a small number of reports regarding the outcomes in PNH patients who received a HID-HSCT and accordingly it is important to compare the results of these transplants with those of a MSD-HSCT. The current study provides encouraging results on the comparison of between HID-HSCT and MSD-HSCT in PNH patients.

In conclusion, this comparative study in PNH patients indicated that outcomes after transplantation using HID were comparable to those using MSD. HID-HSCT should therefore be recommended as a viable alternative for PNH patients with no suitable HLA-matched donor. However, our study was limited by its retrospective design and large-scale, multicenter, cooperative prospective studies are therefore required to confirm our results.

## Supplementary information

Table S1
