# Supplementary material for: Haploidentical vs matched sibling donor transplant for paroxysmal nocturnal haemoglobinuria: A multicenter study
Source: Blood Cancer J. 2022 Jun 24;12(6):92. doi: 10.1038/s41408-022-00682-w (PMC9232486; doi:10.1038/s41408-022-00682-w)
Supplement: Supplementary file 1 — Table S1 [file 41408_2022_682_MOESM1_ESM.docx]

Table S1. Multivariate analysis of favorable factors associated with survival and GVHD

| Outcome | Hazard ratio | P |
| --- | --- | --- |
| OS |  |  |
| MSD (vs. HID) | 1.816 | 0.225 |
| Age | 1.032 | 0.096 |
| Classical PNH (PNH in the setting of another BM disorder) | 1.033 | 0.951 |
| Median time from diagnosis to transplantation | 1.005 | 0.108 |
| Source of graft (BM+PB vs. PB) | 0.620 | 0.365 |
| GFFS |  |  |
| MSD (vs. HID) | 1.992 | 0.080 |
| Age | 1.016 | 0.330 |
| Classical PNH (PNH in the setting of another BM disorder) | 0.887 | 0.778 |
| Median time from diagnosis to transplantation | 1.003 | 0.317 |
| Source of graft (BM+PB vs. PB) | 1.138 | 0.749 |
| Grade 2-4 aGVHD |  |  |
| MSD (vs. HID) | 1.899 | 0.082 |
| Age | 1.033 | 0.082 |
| Classical PNH (PNH in the setting of another BM disorder) | 1.211 | 0.717 |
| Median time from diagnosis to transplantation | 1.005 | 0.069 |
| Source of graft (BM+PB vs. PB) | 0.414 | 0.110 |
| Grade 3-4 aGVHD |  |  |
| MSD (vs. HID) | 1.121 | 0.598 |
| Age | 1.020 | 0.598 |
| Classical PNH (PNH in the setting of another BM disorder) | 0.340 | 0.214 |
| Median time from diagnosis to transplantation | 1.002 | 0.774 |
| Source of graft (BM+PB vs. PB) | 0.383 | 0.406 |
| Total cGVHD |  |  |
| MSD (vs. HID) | 1.922 | 0.113 |
| Age | 1.010 | 0.558 |
| Classical PNH (PNH in the setting of another BM disorder) | 0.605 | 0.256 |
| Median time from diagnosis to transplantation | 1.001 | 0.727 |
| Source of graft (BM+PB vs. PB) | 1.402 | 0.444 |
| Moderate-severe cGVHD |  |  |
| MSD (vs. HID) | 2.312 | 0.192 |
| Age | 1.003 | 0.901 |
| Classical PNH (PNH in the setting of another BM disorder) | 1.131 | 0.880 |
| Median time from diagnosis to transplantation | 1.002 | 0.716 |
| Source of graft (BM+PB vs. PB) | 3.521 | 0.056 |

Abbreviations: GVHD, graft-versus-host disease; OS, overall survival; MSD, matched sibling donor; HID, haploidentical donor; PNH, paroxysmal nocturnal haemoglobinuria; BM, bone marrow; PB, peripheral blood; GFFS, GVHD-free, failure-free survival; aGVHD, acute GVHD; cGVHD, chronic GVHD
